# NTCP Modeling of Late Effects for Head and Neck Cancer: A Systematic Review

**DOI:** 10.14338/20-00092

**Published:** 2021-06-25

**Authors:** Sonja Stieb, Anna Lee, Lisanne V. van Dijk, Steven Frank, Clifton David Fuller, Pierre Blanchard

**Affiliations:** 1 Department of Radiation Oncology, The University of Texas MD Anderson Cancer Center, Houston, TX, USA; 2 Center for Radiation Oncology KSA-KSB, Kantonsspital Aarau, Aarau, Switzerland; 3 Department of Radiation Oncology, University Medical Center–Groningen, Groningen, the Netherlands; 4 Department of Radiotherapy, Gustave Roussy Cancer Campus, Universite Paris-Saclay, Villejuif, France

## Introduction

Head and neck cancer survival rates are increasing as a result of more advanced treatment regimens [1–7]. The increasing number of head and neck cancer survivors stresses the importance of preventing late radiation-induced toxicities, which may persist or occur years after treatment, gravely impacting the quality of life [8]. Owing to the intrinsic properties of particles, proton therapy has the ability to deliver dose more conformal to the tumor, consequently sparing more normal tissue surrounding it [9–11]. While proton therapy arguably can be beneficial for a large proportion of head and neck cancer patients, it is currently still limited in availability and there is a benefit gradient with regard to the estimated toxicities [12, 13]. Introduced by Langendijk et al [14], the model-based approach is a systematic way to identify patients that may benefit most from proton therapy based on the predicted toxicity risk, that is, normal tissue complication probability (NTCP). In other words, NTCP can be considered the individual percentile risk for a patient to develop a certain radiation-associated toxicity (eg, risk of 40% to develop feeding tube dependence).

NTCP-guided treatment decision support requires reliable NTCP models, which generally embody the toxicity's association with organ-at-risk (OAR)-specific dose-volume histogram parameters. The development of (semi)auto-contouring of head and neck OARs has facilitated more time-efficient and robust extraction of dose-volume histogram information, particularly from OARs that are not contoured for clinical planning purposes [15–18]. In addition, clinical factors (eg, chemotherapy, smoking status, age, xerostomia before radiotherapy) can contribute to or interact with the effect of radiation dose in developing radiation-induced toxicities [15–17, 19–21].

The aims of this systematic review were (1) to describe how NTCP models are developed and validated, (2) to perform and present a systematic review of existing NTCP models for photon or proton therapy for head and neck cancers, and (3) to explore and propose future directions, concerning novel NTCP model types, as well as methodologic development.

### Normal Tissue Complication Probability Model Development and Validation

NTCP models are classifiers, meaning they aim to stratify between patients at high risk and low risk for developing a toxicity. In contrast to the historically used Lyman–Kutcher–Burman NTCP models [18, 22, 23], which are only based on a single-dose variable, multivariable logistic regression represents the current modeling preference as shown in the following equation:

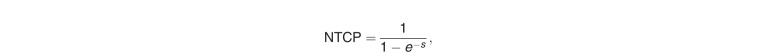
where *s* = β_0_ + β_1_·*variable*_1_ + β_2_·v*ariable*_2_… + β*_n_*·*variable_n_*.

The β_0_ is the intercept coefficient, which is a constant; the other β are the coefficients multiplied by, thus linked to, a specific variable, which could be for example the mean contralateral parotid gland dose or baseline xerostomia complaints.

**Figure 1.**
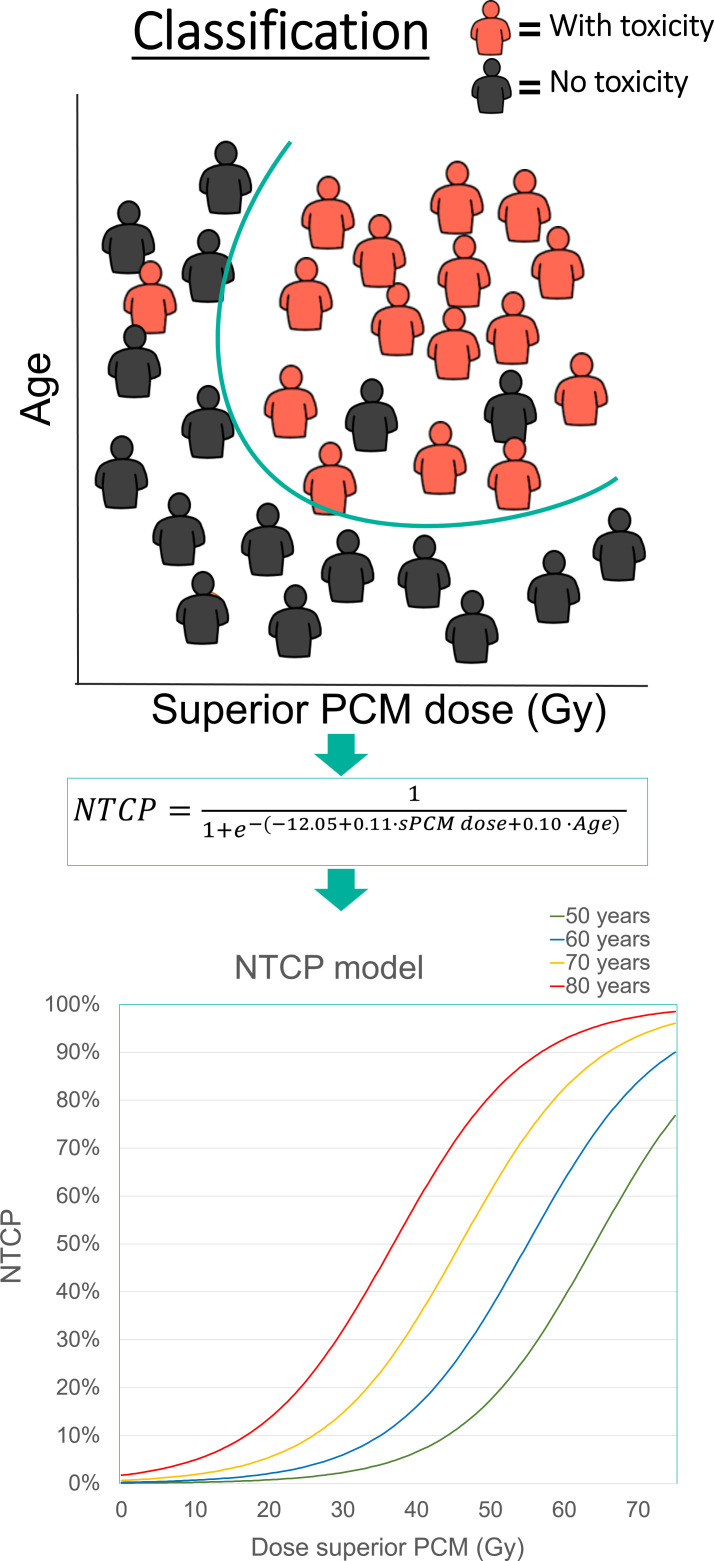
Normal tissue complication probability model optimization.

Model development is performed by “training” on training data to (1) select an optimal set of variables that are significantly associated with the predicted toxicity, and (2) estimate the model coefficients (β) to fit the model to the data. Figure 1 illustrates a hypothetical example of an NTCP classification (ie, the equation); with a so-called loss function, the most optimal distinction is sought to separate patients with and without a toxicity. The linear prediction (*s* in the equation) that is obtained from this optimization process is the basis of the resulting logistic regression NTCP model (visual representation in Figure 1).

One of the commonly used variable selection approaches is the forward (or Step-Up) Stepwise selection, which adds variables to the model, while testing the significance of model improvement. Alternatively, backward Stepwise selection operates in a reverse manner, by entering the full pool of variables to the model and subsequently removing variables. This significant improvement is often tested with a likelihood-ratio test, or alternatively with Akaike Information Criterion. Another popular variable selection method is the L1, or Lasso, regularization where, like backward selection, all variables are presented to the model and by increasing the penalization term lambda, the regularization shrinks the coefficients of the variables. Consequently, the effect size is reduced for the variables that do not contribute to the model, and thereby procedurally excluding variables when their coefficient approaches zero [22, 24].

The inherent aim of developing an NTCP model is that the model is “generalizable” to new data. Internal validation can improve the robustness of the NTCP model by iteratively subsampling the training data and repeating the entire training process, by which robust variable selection and coefficient can be obtained. The most common variants are cross-validation and bootstrapping [25]. The Transparent reporting of a multivariable prediction model for individual prognosis or diagnosis statement group has excellent modeling guidelines, and is a supply source to consult for consistent train and test modeling [26, 27].

Ultimately, the highest form of validation is obtained by performing external validation on data from multiple centers. Following the transparent reporting of a multivariable prediction model for individual prognosis or diagnosis statement classification [28] and the Dutch proton indication protocol [29], Table 1 details the different levels of evidence for NTCP models.

**Table 1.** Level of evidence for NTCP-models, based on TRIPOD [28] and NVRO [29].

**Level**	**Description**
1a	NTCP model that is externally validated on an independent multi-institute dataset with different treatment modality (proton therapy)
1b	NTCP model that is externally validated on independent data from another institute
2a	NTCP model trained and externally validated on nonrandomly split of single-center data
2b	NTCP model trained and externally validated on randomly split of single-center data
3	NTCP model developed with internal validation
4a	Multivariable NTCP model without internal/external validation
4b	Univariable NTCP model without internal/external validation

**Abbreviations:** NTCP, normal tissue complication probability; TRIPOD, transparent reporting of a multivariable prediction model for individual prognosis or diagnosis; NVRO, Nederlandse Vereniging voor Radiotherapie en Oncologie.

## Materials and Methods

**Figure 2.**
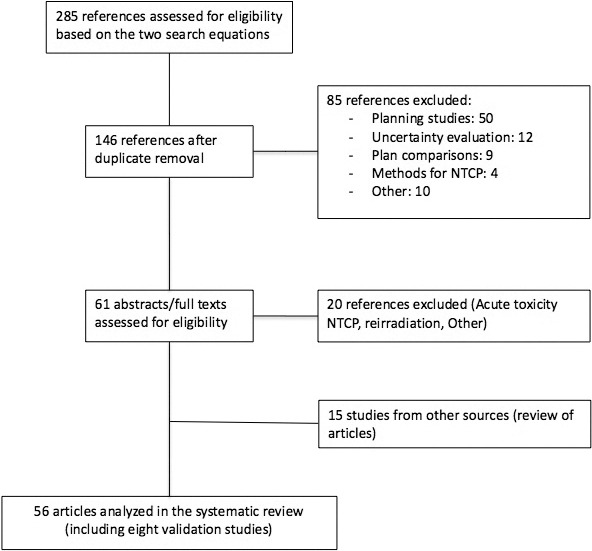
PRISMA flow chart.

For the systematic review of existing NTCP models, the inclusion criteria were NTCP models that predict late toxicity after radiotherapy in head and neck cancer patients. The article search was performed on PubMed, by 2 board-certified radiation oncologists (PB and SS), using the following 2 search equations: “([‘head neck neoplasms'(MeSH Terms)] AND [(NTCP)[MeSH Terms] or ‘normal tissue complication'])” and “(‘head neck neoplasms'[MeSH Terms] AND ‘normal tissue complication probability').” After removal of duplicates the references were reviewed by the 2 investigators (PB and SS) and discrepancies were discussed with a third radiation oncologist (AL). The flow chart is presented in Figure 2 [30]. Most articles excluded were using NTCP models to quantify the clinical meaning of differences according to variation in treatment planning systems. Sixty-one articles were retrieved that presented NTCP modeling. Twenty were finally excluded because of focus on acute toxicity, reirradiation, or because of focus on planning. The overall quality of the NTCP models was ranked as poor, fair, good, or excellent according to criteria pertaining to endpoint definition and collection, sample size, and data analysis/validation.

### Review of Current Normal Tissue Complication Probability Models

In total, 48 studies and 8 validation studies were found with NTCP modeling to predict late radiation-associated toxicity after irradiation of tumors in the head and neck area (Figure 2, **Supplemental Table**). The majority of these studies developed models for the prediction of late xerostomia (n = 25), followed by dysphagia/feeding tube dependency/esophageal stricture (n = 7), and brain necrosis/nerve palsy (n = 6) fewer studies investigated prediction of toxicities, such as hypothyroidism (n = 3), hearing loss/tinnitus (n = 3), trismus (n = 2), taste impairment (n = 1), dry eye (n = 1), laryngeal edema (n = 1), and hypopituitarism (n = 1) (2 studies developed models for 2 different toxicities, respectively). No study was found to report on models predicting late fibrosis/skin changes, osteoradionecrosis, hoarseness, aspiration/choking, weight loss, carotid artery calcification, fatigue, or second malignancies. The mean dose parameters that were generally selected for late toxicities were related to glandular structures (eg, parotid, submandibular, or thyroid gland) and muscular structures (eg, superior pharyngeal constrictor muscles or masseter). The maximum dose, or robust representation (eg, D_1cc_) were selected in the final models of severe neurologic injury (eg, brain necrosis or nerve palsy), while this was not the case for hearing loss. Some of the final NTCP models included clinical variables, that is, for xerostomia, the baseline scores for dry mouth showed to be of importance [12, 20], while for tube feeding weight loss and chemotherapy were included in the final model [31]. Nevertheless, most studies did not select clinical variables in the NTCP model, which may be owing to most being Lyman–Kutcher–Burman models, the relatively small number of patients, or that potential variables were not included in the variable selection pool (eg, many studies did not include baseline symptom scores).

While many of the included studies were based on low patient numbers, 28 studies (29 models) (58%) had cohorts of over 100 patients. In addition, most studies lacked internal and/or external validation as follows: only 9 studies performed internal, 8 external validation, and 5 studies both, internal and external validation. Ten studies included patients treated with nonadvanced treatment techniques only, and in 10 other studies at least a certain proportion of patients were included with 3-dimensional conformal radiation therapy. Only 4 studies (5 models) included patients with proton therapy. With these considerations, an overview of the most complete studies is depicted per symptom category in Table 2. Most of the models were rated as good or fair; none of the models was rated as excellent due to the reasons described above.

**Table 2.** Selection of the best available NTCP models per symptom category in head and neck cancer patients.

**Author**	**Patients, n, (OARs)/events**	**Advanced RT techniques only**	**Endpoint ≥ 12 m post-RT**	**Model**	**Variables included in final model**	**Prospective toxicity collection**	**Prediction probability**	**Validation**	**Others**	**Overall quality**
Xerostomia										
Beetz 2012 [20]	17/83	Yes (IMRT)	No (6 m)	Logistic regression with bootstrapping	Xerostomia: Dmean PG contra, BL xerostomia Sticky saliva: Dmean SMG contra, Dmean SLG both, Dmean soft palate	Yes	AUC 0.68 (xerostomia), 0.70 (sticky saliva)	Yes (internal)		Good
Lee 2014 [32]	158/52	Yes (IMRT)	Yes (3 m and 12 m)	LASSO logistic regression with bootstrapping	12 m: Dmean PG ipsi/contra	Yes	AUC 12 m: 0.98 (HNSCC), 0.96 (NPC)	Yes (internal)		Good
van Dijk 2017 [33]	249/63 (sticky saliva)/100 (xerostomia)	No (IMRT, 3DCRT, VMAT)	Yes (1 y)	LASSO multivariate logistic regression	Xerostomia: Dmean PG contra, BL xerostomia, SRE GLRLM PG contra; Sticky saliva: Dmean SMG (both), BL sticky saliva, max HU both SMG	Yes	AUC xerostomia ± imaging biomarker 0.77 and 0.75, sticky saliva ± imaging biomarker 0.77 and 0.74	Yes (internal)	Limited added value of imaging biomarkers	Good
Dysphagia										
Christianen 2012 [19]	354/NA	No (3DRT, IMRT)	No (6 m)	Logistic regression	Dmean SPC, Dmean supraglottic larynx	Yes	AUC 0.80	Yes (external)		Good
Feeding tube dependency										
Wopken 2014 [31]	355/38	Yes (IMRT)	No (6 m), but clinically relevant	Logistic regression	T stage, weight loss, accelerated RT, chemo, Cetuximab, Dmean inf. PCM, Dmean PG contra, Dmean cricopharyngeal muscle	Yes	AUC 0,88	Yes (internal)		Good
Dysgeusia										
Sapir 2016 [34]	73/26	Yes (IMRT)	No (3 m)	LKB	Dmean oral cavity	Yes	NA	No	Oral cavity as OAR	Fair
Esophageal stricture										
Mavroidis 2003 [35]	82/26	No (3DCRT)	No 1–40 m (median 7 m)	LKB	Dmean esophagus	No	ROC = 0.84, X^2^ test = 0.95	No		Poor
Brain necrosis										
Wang 2019 [36]	749/38	Yes (IMRT)	No (3.5–75 m, median 49 m)	Lasso binary regression	D0.5 cc and D10 selected for final model	No	AUC 0.68 (testing set)	Yes (internal)		Good
Zeng 2015 [37]	351/29	Yes (IMRT)	No (6–100 m, median 76 m)	Logistic regression	D_1cc_	No	NA	No		Fair
Nerve palsy										
Chow 2019 [38]	330 nerves/46	Yes (IMRT)	No (min. FU 6 m, median 8.1 y)	Logistic regression	D_1cc_	No	AUC 0.83	No	False high rate of palsies: min. FU all patients 6 m/healthy control 8 y	Fair
Trismus										
Morimoto 2019 [39]	132/30	No (3DCRT, IMRT (percentage unclear)	No (6 m)	Logistic regression	Dmean TMJ contra, max. intercisial opening at BL	Yes	*P* = .182 (Hosmer and Lemeshow test)	No	Collinearity check; acc. fx in 95 patients	Good
Lindblom 2014 [40]	121/50	No (3DCRT, IMRT)	Yes for MID (21–127 m, median 66 m), unclear for QoL scores	Logistic regression	Different models with different variables studies; best fit for ipsi masseter for both endpoints	No (MID), yes (QoL)	0.77 and 0.73 for model with endpoint MID and QoL, respectively and ipsi masseter as variable	Yes (internal)	70 patients with acc. fx	Good
Hypothyroidism										
Rønjom 2013 [41]	203/35	Yes (IMRT)	Yes (1 y, 2 y)	Logistic regression	Dmean thyroid gland, thyroid gland volume	No (but objective criterion)	NA	No		Good
Hearing loss										
Marzi 2015 [42]	280 ears/73	Yes (PT)	Yes (median FU 26 m)	LKB	Dmean inner ear	Yes	AUC 0.86	No		Fair
Tinnitus										
Lee 2015 [43]	422 ears/49	Yes (IMRT)	Yes (51 m, range 36– 77 m)	LKB Logistic regression	Dmean cochlea ipsi	No	LKB: 0.76, Logistic: 0.76	No	Influence of chemotherapy not accounted for	Fair
Dry eye										
Bhandare 2012 [44]	78/40	No (EBRT 1996–2000)	No (mean 0.9 y)	Logistic regression with bootstrapping	Dmax lacrimal gland	No	NA	No		Fair
Laryngeal edema										
Rancati 2009 [45]	48/25	Yes (IMRT)	Yes (15 m)	Lyman Logit	Dmean larynx	No (but objective criterion)	NA	No		Fair
Hypopituitarism										
Marzi 2015 [42]	103/45	Yes (PT)	Yes (median 26 m)	LKB	Dmean pituitary gland	Yes	AUC 0.86	No		Fair

**Abbreviations:** NTCP, normal tissue complication probability; OAR, organ at risk; RT, radiotherapy; prosp., prospective; IMRT, intensity modulated radiation therapy; Dmean, mean dose; PG, parotid gland; Contra, contralateral; BL, baseline; SMG, submandibular gland; SLG, sublingual gland; AUC, area under curve; ipsi, ipsilateral; HNSCC, head neck squamous cell cancer; NPC, nasopharyngeal cancer; 3DCRT, 3-dimensional conformal radiotherapy; VMAT, volumetric modulated arc therapy; SRE GLRLM, short run emphasis gray level co-occurrence; HU, Hounsfield unit; NA, not assessed; PCM, Pharyngeal Constrictor Muscle; LKB, Lyman–Kutcher–Burman; ROC, receiver operator curve; min, minimum; FU, follow-up; TMJ, temporomandibular joint; acc. fx, accelerated fractionation; MID, maximal interincisal distance; QoL, quality of life; PT, proton therapy; EBRT, external beam radiotherapy.

### Normal Tissue Complication Probability Models for the Selection of Proton Therapy

The patients that benefit the most from proton therapy would ideally be identified with NTCP models that are trained on larger intensity modulated radiation therapy/volumetric modulated arc therapy–treated patient cohorts and subsequently validated in proton therapy patients. Unfortunately, these models do not currently exist (**Supplemental Table**). Two studies have developed NTCP models predicting brain necrosis in a proton therapy cohort only [46, 47]. Nevertheless, even with current challenges of determining the generalizability of NTCP models for both treatment modalities, NTCP differences (ΔNTCP) are still anticipated to be a better representation of the clinical importance (ie, expected clinical symptom burden reduction) than direct dose-volume histogram parameter differences. Consequently, ΔNTCP are considered a better marker to identify patients that will benefit most from proton compared with photon therapy. This is the foundation of the model-based approach [14], where validated models can serve as a tool to assess the late toxicity ΔNTCP between a photon and proton therapy plan. If the NTCP or ΔNTCP of a specific or multiple OARs exceed a clinically relevant toxicity-specific threshold, proton therapy might be the preferred treatment modality for that patient. Herein lies a weighting factor of the severity of toxicities, and some toxicities may be negligible of influence, such as hypothyroidism, which is treatable with medical substitution.

A recently published paper by Tambas et al [48] describes a practical institutional workflow of proton-photon plan comparison decision-making in a patient cohort from the University Medical Center Utrecht. Patients who have a ≥ 10% higher risk of developing grade 2+ xerostomia, grade 2+ dysphagia, ≥ 5% risk for feeding tube dependency, or a ≥ 15% higher risk in the combination of those toxicities with photon compared with proton radiotherapy will be considered for proton therapy. The models used for this estimate [19, 20, 31] are predicting the toxicities at 6 months postradiotherapy and are externally validated. There is still a need to develop models for later time points, as Janssens et al [49], for example, showed a decrease in incidence of xerostomia from over 40% at 6 months postradiotherapy to approximately 25% 24 months postradiotherapy.

Despite the usefulness of NTCP models in guidance of patient selection for proton therapy, the final decision regarding the treatment modality may still be influenced by individual factors of the patient (ie, cost, travel, scheduling) and proton therapy capacity; thus, clinical prioritization and insurance issues should ultimately be left to the discretion of the patient and treating physician, taking into account the patient's goals and limitations of the NTCP models.

### Normal Tissue Complication Probability Model Considerations

While dose-volume histogram parameters extraction comes with its challenges (eg, OAR definitions [50, 51], corrupt dose information, treatment plan system differences), the limiting factor in NTCP model data accumulation is the availability of qualitative toxicity data. While standardized follow-up programs are slowly being introduced [52, 53], many radiotherapy centers do not systematically collect toxicity information, leading to sparsely available and retrospectively collected data. Introduction of proton therapy has provoked improved toxicity collection, owing to the need to show the beneficial OAR sparing with proton therapy compared with photon therapy. Therefore, NTCP model development based on big data cohort is expected in years to follow.

The inclusion of a wide spectrum of different head and neck tumor subsites and stages into the modeling process can improve the model performance, as it provides a greater variability in dose administered to specific OARs. The downside of wide-inclusion criteria is that therapy patients receive may differ, such as receipt of induction and/or concurrent chemotherapy. While chemotherapy as a variable is often not selected in the final model, it may in fact affect the toxicity development, but may be too rudimentarily examined (**Supplemental Table;** eg, chemotherapy yes/no, no number of cycles), not adequately represented (eg, adaptation of agent dosing), or without sufficient patient numbers to measure the effect. Furthermore, the biological dose of OARs might be different according to the corresponding alpha/beta ratio, which might translate to a different toxicity development; for example, in the case of hypofractionated/accelerated radiotherapy treatment schedules. Current NTCP models are not designed to include altered fractionation, nevertheless some studies investigated the fractionation effect [31, 39, 54] (**Supplemental Table**), whereas others did not [38]. Other studies showed the potential of improving NTCP models with the addition of baseline imaging biomarkers [33, 55, 56], which represent patient-specific tissue characteristics. These image biomarkers in NTCP models are still novel and are hampered by the availability of magnetic resonance imaging or positron emission tomography–computed tomography for treatment planning in some institutions. None of the studies, however, has taken into account if the patient had major surgery before radiotherapy. For example, if a salivary gland had to be removed because of tumor infiltration, this adds a significant risk to the development of xerostomia.

### Future Directions

Radiation-induced toxicities are often difficult to predict before treatment, as they are patient-specific, complex, interdependent, and nondeterministic. Currently, the vast majority of NTCP models rely on minimal input variables, making the estimated risk of radiation-induced side effects relatively simplistic. New modeling approaches, such as machine and deep learning, have the capacity to handle high-dimensional data, such as imaging, spatial localization of radiation dose, and potentially alterations in intertoxicity interactions over time. Nevertheless, there will always remain patient-specific factors, that are difficult to account for in modeling, like genetic susceptibility to radiotherapy toxicity, patient adherence with medication and abstaining from smoking/alcohol, tolerability of concurrent chemotherapy or targeted therapy, unexpected treatment breaks, and so on.

With modern photon techniques, like intensity modulated radiation therapy and volumetric modulated arc therapy, there is increased heterogeneity of the dose within OARs [57], which can improve the NTCP, albeit with higher integral dose [58]. In other words, the need to include the information from the entire dose-volume histogram curve or maybe even the full 3-dimensional spatial distribution of dose may be needed to improve the performance of NTCP models in the current and future era. Moreover, the applicability of intensity modulated radiation therapy studies to patients receiving proton therapy is limited as the relative biological effectiveness function needs to be considered. Indeed, it has been demonstrated that relative biological effectiveness varies along the beam path and depending on where the Bragg peaks are distributed, and this could lead to increased toxicity. In a series of 34 children treated using proton therapy, Peeler and colleagues [59] showed that showed T2-FLAIR hyperintensity was dependent on linear energy transfer dose, which indicated a possible variable biological dose effectiveness with clinical implications. For photon models to be incorporated to proton therapy, relative biological effectiveness–based weighting would ideally be needed although the validation of such models will require a lot of prospectively followed patients. Furthermore, range uncertainty in proton therapy needs to be considered and anatomic changes, such as paranasal sinus filling, swelling of irradiated tissue, or weight loss under therapy can lead to a lack of robustness in proton plans. This effect is minimized in photon therapy but can become quite significant in proton therapy, especially if OARs are lying just behind the target/Bragg peak, and can vary from institution to institution and with differing forms of proton delivery technique (passively beam scattered protons versus intensity modulated proton therapy). It has been previously shown that NTCP models developed for photon therapy did retain discriminatory properties for proton therapy and could hence be used to select patients [60]. Furthermore, the conformality afforded by proton therapy decreases concerns such that an NTCP model can reliably score a radiation plan for both photons and protons [61, 62].

As dedicated NTCP modeling for particle therapy is being developed, the issue of modeling for reirradiation becomes particularly salient as many centers are using proton therapy in this setting. The specific challenges in these cases include incorporation of different fractionation schemes, accounting for normal tissue recovery and dose-volume overlap [32].

The various challenges with developing adequate models are numerous but one important step with the right study design is through a multicenter approach. Van den Bosch and colleagues [58] from the Netherlands have discussed that consistent definitions of predictor and outcomes variables as well as use of standardized scoring systems are needed to reduce heterogeneity of data. Uniform delineation guidelines should also be used to improve consistency of OARs. To achieve this aim, a multicenter prospective trial is needed so that the final models can be applicable in a variety of settings. Currently, there are 2 ongoing head and neck multicenter, randomized trials (MD Anderson IMPT vs IMRT Trial, NCT01893307 [63] and UK TORPEDO Trial, ISRCTN16424014 [64]) that have the potential to validate and expand these NTCP models to achieve a more robust prediction of clinical benefit for appropriate patient selection and access to proton therapy. Novel NTCP modeling and validation has significant health policy considerations for future head and neck cancer patients, and buy-in from the scientific community is needed with government level funding to achieve this aim.

However, NTCP modeling will always be an estimation of risk, rather than a real prediction of toxicity. With all the techniques described above (machine/deep learning, multicenter validation, etc.) models will significantly improve in the next decades and can help as a tool to select patients most likely to benefit from proton therapy or to define organs at risk, which should be spared with higher priority. However, these models will never incorporate all factors, which influence the outcome of every single patient and should therefore never replace the physician's clinical judgement.

In conclusion, we presented a systematic review of NTCP modeling studies performed for head and neck cancers. These studies have improved in quality over time and will help define how to select a treatment modality for a given patient. Future investigations with large number of cases are needed to apply NTCP models in practice and should also include reduction in acute toxicity (ie, mucositis) to aid with improved selection of patients.
