# Supplementary material for: NTCP Modeling of Late Effects for Head and Neck Cancer: A Systematic Review
Source: Int J Part Ther. 2021 Jun 25;8(1):95–107. doi: 10.14338/20-00092 (PMC8270107; doi:10.14338/20-00092)
Supplement: Supplementary file 1 [file ijpt-08-01-26_s01.pdf]

Supplementary Material

Supplementary Table I: Overview of NTCP models in head and neck cancer patients to predict toxicity >=6 months from radiotherapy

Author, year Group	Number of patients	Tumor entity	RT tech-nique	Endpoint	Model	Variables studied/ included in final model	Overall quality
**Xerostomia**							
van Dijk, 2016 [1]	249	HNC (L, OP, HP, NP, OC, CUP)	3DCRT (9%), IMRT (87%), VMAT (4%)	Mod./sev. xerostomia and sticky saliva (EORTC QLQ H&N35 two highest scores) at 1y post-RT	LASSO multivariate logistic regression<br><br>Model parameters (multiple models, see Table 3 of article)	Studied: sex, age, overall stage, TNM stage, tumor site, RT technique, systemic treatment, weight, lenghth, BMI, BL xerostomia, BL sticky saliva, and various imaging variables (n=130)<br><br>Included:<br>Xerostomia: Dmean PG contra, BL xerostomia, SRE GLRLM (Parotid imaging biomarker, unclear if ipsi/contra/both)<br>Sticky saliva: Dmean SMG (unclear if ipsi / contra / both), BL sticky saliva, max HU SMG (Imaging biomarker, unclear if ipsi/contra/both)	Good<br>- Endpoint >=12m from RT start: yes<br>- Advanced RT techniques only, no reirradiation: no<br>- N pts. / N events: 249/ 63 (sticky saliva), /100 (xerostomia)<br>- Validation: yes (internal cross validation/bootstrapping)<br>- Prospective toxicity collection: yes<br>- Prediction probability: AUC 76xerostomia with and without imaging biomarker 0.77 and 0.75, sticky saliva with and without imaging biomarker 0.77 and 0.74<br>- Others: Limited added value of imaging biomarkers, difficult to assess
Dijkema, 2010 [2]	222	HNC (OP, L, OC, NP, Others, NC, HP, SG, CUP)	IMRT	Stimulated parotid flow rate <25% of BL flow rate grade 4 (RTOG/EORTC) xerostomia at 1y post-RT	LKB<br><br>TD50=39.9Gy, m=0.40, n=1	Studied: sex, age, tumor site, overall stage, def. vs. postop. RT, recurrence Dosimetric parameters of parotid mean dose; both glands<br><br>Included: Dmean PG	Good<br>- Endpoint >=12m from RT start: yes<br>- Advanced RT techniques only, no reirradiation: no<br>- N pts. / N events: 222 (384 PGs)/NA<br>- Validation: yes (external, Houweling [3])<br>- Prospective toxicity collection: yes<br>- Prediction probability: NA
Dijkema, 2008 [4]	221	HNC (OP, L, OC, NP, Others, NC, HP, CUP)	3DCRT (71%), IMRT (29%)	Stimulated parotid flow rate <25% of BL flow rate at 6w, 6m, 1y post-RT	LKB<br><br>1y, RCT: TD50=40 Gy, m=0.46, n=1;<br>1y, IMRT: TD50=38, m=0.33, n=1	Studied: Dmean PG (unclear if ipsi, contra or both included)<br><br>Included: Dmean PG (unclear if ipsi, contra or both included)	Fair<br>- Endpoint >=12m from RT start: yes<br>- Advanced RT techniques only, no reirradiation: no, but separate analysis for 3DRT and IMRT<br>- N pts. / N events: 221/ 90<br>- Validation: yes (external, Houweling [3])<br>- Prospective toxicity collection: yes<br>- Prediction probability: NA<br>- Others: Unclear if Dmean PG ipsi / contra / both
Beetz, 2012 [5]	178	HNC (OC, OP, L/HP, NP, PNS)	IMRT	Mod./sev. xerostomia at 6m and sticky saliva after completion of RT	Logistic regression<br><br>NTCP = (1 + e^-s)^-1, where<br>s= -1.443 + (Dmean PG contra x 0.047) + (BL xerostomia score x 0.720)	Studied: sex, age, site, T stage, N positivity, bilat. neck RT, chemo, Cetuximab, Dmean PG contra, Dmean SMG contra, Dmean SLG both<br><br>Included: Dmean PG contra, Dmean? SMG contra, BL xerostomia, Dmean	Good<br>- Endpoint >=12m from RT start: no (assessed 6 months)<br>- Advanced RT technioLues only, no reirradiation: yes<br>- N pts. / N events: 178/83<br>- Validation: yes (internal bootstrapping and external, Blanchard [6])<br>- Prospective toxicity collection: yes

Author, year Group	Number of patients	Tumor entity	RT tech-nique	Endpoint	Model	Variables studied/ included in final model	Overall quality
						SLG both, Dmean soft palate	- Prediction probability: yes; AUC 0.68 for xerostomia and AUC 0.70 for sticky saliva (acute tox)
Beetz, 2012 [7] (similar to study above but looked at 3DCRT and toxicity at 6m)	167	HNC (OP, NP, HP, L, misc)	3DCRT	Mod/sev xerostomia (EORTC QLQ-HN35) at 6m post-RT	Logistic regression<br><br>NTCP = (1 + e^-s)^-1, where<br>s= -5.27 + (Dmean PG x 0.066) + age x 0.050) + (baseline xerostomia score x 0.916)	Studied: sex, age, chemo, T stage, N stage, site, bilat neck RT, medical centre<br><br>Included: Dmean PG both, Dmean SMG both, Dmean SLG both	Good<br>- Endpoint >=12m from RT start: no (assessed 6 months)<br>- Advanced RT technioLues only, no reirradiation: no<br>- N pts. / N events: 167/87<br>- Validation: - Validation: yes (internal boostrapping and external Beetz [8])<br>- Prospective toxicity collection: yes<br>- Prediction probability: AUC 0.82 for xerostomia, AUC 0.84 for sticky saliva
Van Dijk, 2018 [9]	161	HNC (OP, L, HP, NP, OC)	IMRT (90%), VMAT (10%)	Mod./sev. xerostomia (EORTC QLQ H&N 35) at 12m post-RT	Lasso logistic regression<br><br>NTCP = 1(1 – e^-s)<br><br>a) Intensity PET IBM:<br>s= 0.984 + 0.048 x Dmean PG contra + 1.402 x BL xerostomia – 1.527 x P90 PG<br>b) Textural PET IBM:<br>s= -2.598 + 0.051 x Dmean PG contra + 1.479 x BL xerostomia – 0.880 x (LRHG3E(PG) – 201.24 / 177.05)	Studied: Dmean PG contra, BL xerostomia, 24 intensity PET biomarkers: SUVmean, min, max PG contra, 66 textural features (unclear if ipsi/contra/both)<br><br>Included: BL xerostomia, Dmean PG contra, P90, LRHG3E (both imaging biomarkers, unclear if ipsi/contra/both)	Poor<br>- Endpoint >=12m from RT start: yes<br>- Advanced RT techniques only, no reirradiation: yes<br>- N pts. / N events: 161/60<br>- Validation: yes (internal cross validation/bootstrapping)<br>- Prospective toxicity collection: yes<br>- Prediction probability: AUC: a) 0.77, b) 0.77<br>- Others: “Halo” of PET positive level II lymph nodes in PG not accounted for
Gabrys, 2017 [10]	153	HNC (OP, HP/L, NP, Others)	IMRT (24%), Tomotherapy (76%)	G1+/G2+ xerostomia (mainly CTCAE grading) 6m (+/-3m), 12m (+/-3m) post-RT start	LKB<br><br>G1+, 12m, PG contra: TD50=12.0 Gy, m=0.61, n=1; G1+, 12m, PG both: TD50= 13.6Gy, m=0.61, n=1;<br>G2+, 12m, PG contra: TD50=51.6Gy, m=0.60, n=1;<br>G2+, 12m, PG both: TD50=64.8Gy, m=0.60, n=1	Studied: Dmean PG contra and both PGs<br><br>Included: Dmean PG contra and both PGs	Good<br>- Endpoint >=12m from RT start: yes<br>- Advanced RT techniques only, no reirradiation: yes<br>- N pts. / N events: 153/120 (G1+) and 27 (G2)/138 (6m)<br>- 83 (G1+) and 15 (G2)/102 (12m)<br>- Validation: no<br>- Prospective toxicity collection: no<br>- Prediction probability: AUC G1+ xerostomia: 0.69 – 0.76, G1+ 0.49 – 0.56 (G2+)<br>- Others: Satisfactory model for G1+ only, which might be less relevant
Roesink, 2004 AL [11]	108	HNC (larynx, OC, OP, NC, NP, HP, CUP)	3DCRT	Salivary excretion fraction (SEF) of <45% of BL flow at 6w and 1y post-RT	Lyman<br><br>1y, SEF<25%: TD50=52Gy, m=0.42, n=1;<br>1y, SEF<35%: TD50=47Gy, m=0.48, n=1;<br>1y, SEF<45%: TD50=43Gy, m=0.53, n=1;<br>1y, SEF<55%: TD50=40Gy, m=0.59, n=1;<br>1y, flow ratio<25%: TD50=39Gy, m=0.45, n=1	Studied: sex, age, tumor site, T stage, N stage, surgery before RT, Dmean PG both<br><br>Included: salivary excretion fraction (SEF) single PG (unclear which side)	Fair<br>- Endpoint >=12m from RT start: yes<br>- Advanced RT techniques only, no reirradiation: no<br>- N pts. / N events: 1y: 132 glands/93<br>- Validation: yes (external, Houweling [3])<br>- Prospective toxicity collection: yes<br>- Prediction probability: NA<br>- Others: unclear which PG included in model
Lee, 2014 [12]	236	HNC (NP, OC, OP, HP, L, Others)	IMRT	Mod./sev. Xerostomia (EORTC QLQ H&N35 scores	LASSO logistic regression<br>NTCP = (1 + e^-s)^-1<br><br>HNSSC: 12m, PG ipsi: TD50=38.0 Gy,	Studied: 13 (NPC) – 15 (HNSCC) clinical factors, Dmean PG ipsi and contra	Good<br>- Endpoint >=12m from RT start: yes<br>- Advanced RT techniques only, no reirradiation: yes<br>- N pts. / N events: 12m: 143 / 52

Author, year Group	Number of patients	Tumor entity	RT tech-nique	Endpoint	Model	Variables studied/ included in final model	Overall quality
				66 – 100) at 3m and 12m post-RT	y=1.17, PG contra: TD50=25.4 Gy, y=2.41;<br>NPC: 12m, PG ipsi: TD50=42.8 Gy, y=4.35, PG contra: TD50=40.0 Gy, y=3.96	Included (12m):Dmean PG ipsi / contra	- Validation: yes (internal cross validation with boostrapping)<br>- Prospective toxicity collection: yes<br>- Prediction probability: AUC 12m: 0.98 (HNSCC), 0.96 (NPC)
Lee, 2014 [13]	206	HNC (NP, OC, OP, HP, L, Others)	IMRT	G3+ xerostomia (EORTC QLQ H&N35 scores 66 - 100) at 3m and 12m post-RT	LASSO logistic regression<br><br>NTCP = (1 + e^-s)^-1, where s=-12.045+(Dmean PG ipsi x 0.176) + (education x corresponding coefficient) + (Dmean PG contra x 0.121) + (smoking x 0.993) + (T stage x corresponding coefficient) + (BL xerostomia x 0.898) + (alcohol x 0.913) + (family history x -0.871) + (N stage x 1.034)	Studied: sex, age, T stage, N stage, overall stage, SIB vs. sequential RT, chemo, smoking, alcohol, education, family status, family history, financial status, BL xerostomia, Dmean PG ipsi, Dmean PG contra<br><br>Included: T stage, N stage, chemo, smoking, alcohol, education, family history, BL xerostomia, Dmean PG ipsi, Dmean PG contra	Poor<br>- Endpoint >=12m from RT start: yes<br>- Advanced RT techniques only, no reirradiation: yes<br>- N pts. / N events: 12m: 128/43<br>- Validation: yes (internal cross validation)<br>- Prospective toxicity collection: yes<br>- Prediction probability: AUC 0.87<br>- Others: uncommon variable selection
Lee, 2012 [14]	32	HNC (OC, NP, OP, L, PG)	IMRT	Stimulated salivary excretion fraction (SEF) <=45% of BL flow and mod./sev. Xerostomia (EORTC QLQ-H&N35) at 1y	LKB<br><br>1y, SEF: TD50=43.6Gy, m=0.18, n=1<br>1y, QoL: TD50=44.1Gy, m=0.11, n=1	Studied: age, tumor site, surgery, chemo, Dmean PG (unclear if ipsi, contra or both)<br><br>Included: Dmean PG (unclear if ipsi, contra or both)	Poor<br>- Endpoint >=12m from RT start: yes<br>- Advanced RT techniques only, no reirradiation: yes<br>- N pts. / N events: 12m: 31/5 (SEF), 31/6 (QoL)<br>- Validation: yes (internal)<br>- Prospective toxicity collection: yes<br>- Prediction probability: AUC 0.75 (SEF), 0.75 (QoL), Hosmer-Lemeshow test X2=10.2 (p=0.24, SEF), X2=7.76 (0=0.46, QoL)
Roesink, 2001 [15]	108	HNC (L, OC, OP, NC, NP, HP, CUP)	3DCRT	Stimulated parotid flow rate <25% of the preRT flow rate at 6w, 6m, 1y post-RT	Lyman<br><br>6m: TD50=35, m=0.46, n=1;<br>1y: TD50=39, m=0.45, n=1	Studied: sex, tumor site, T stage, N stage, mean parotid dose<br><br>Included: Dmean single PG (unclear which side)	Fair<br>- Endpoint >=12m from RT start: yes<br>- Advanced RT techniques only, no reirradiation: no<br>- N pts. / N events: 108/66<br>- Validation: yes (external, Houweling [3])<br>- Prospective toxicity collection: yes<br>- Prediction probability: NA<br>- Others: unclear which PG included in the model
Hey, 2009 [16]	97	HNC (OP, L, OC, NP, Others, NC, HP, CUP)	3DCRT	Stimulated parotid flow rate <25% of BL flow rate at 4w and 6m post-RT	LKB<br><br>6m, RCT: TD50=32.1 Gy, m=0.59, n=1;<br>6m, RT: TD50=39.6 Gy, m=0.33, n=1	Studied: Dmean both PGs<br><br>Included: Dmean both PGs	Fair<br>- Endpoint >=12m from RT start: no<br>- Advanced RT techniques only, no reirradiation: no<br>- N pts. / N events: 97/NA<br>- Validation: no<br>- Prospective toxicity collection: yes<br>- Prediction probability: NA<br>- Others: Dmean of both PGs used as variable
Eisbuch, 1999 [17]	88	HNC + skin (OP, SG, OC, skin, HP,	3DCRT	Salivary flow rate <=25% preRT flow at 12m post-RT	Lyman<br><br>TD50=28.4Gy, m=0.18, n=1	Studied: sex, age, tumor site, overall stage, chemo, surgery preRT, comorbidities, medications, salivary flow rates both glands	Fair<br>- Endpoint >=12m from RT start: yes<br>- Advanced RT techniques only, no reirradiation: no<br>- N pts. / N events: 12m: 96 glands/37

Author, year Group	Number of patients	Tumor entity	RT tech-nique	Endpoint	Model	Variables studied/ included in final model	Overall quality
		CUP, L, sinus, glomus)				Included: Dmean both PGs	- Validation: yes (external, Houweling [3])<br>- Prospective toxicity collection: no<br>- Prediction probability: NA
Van Dijk, 2018 [18]	68 (+25 ext. validation)	HNC (OP, L, HP, NP, OC, Others)	IMRT (88%), VMAT (12%)	Mod/sev. xerostomia (EORTC QLQ H&N 35, MSKCC questionnaire) at 12m post-RT	Logistic regression<br><br>NTCP = (1 + e^-s)^-1,<br>where s= -6.09 + Dmean SPC x 0057 + Dmean supraglottic larynx x 0.037)	Studied: BL xerostomia, Dmean both PGs<br>Intensity and textural MRI biomarkers on T1 (n=64)<br><br>Included: Dmean both PGs, BL xerostomia, P90 (unclear if ipsi/contra/both)	Good<br>- Endpoint >=12m from RT start: yes<br>- Advanced RT techniques only, no reirradiation: yes<br>- N pts. / N events: 68 / 34<br>- Validation: yes (internal bootstrapping and external)<br>- Prospective toxicity collection: no<br>- Prediction probability: AUC 0.83<br>- Others: Diff. endpoint for training and test cohort; Dmean to both PGs as variable; unclaer if P90 of ipsi, contra, or both PGs
Marzi, 2009 [19]	59	HNC I-IV (NP, OP, HP, OC, CUP)	IMRT	G3+ xerostomia (RTOG) at 3m, 6m, 12m post-RT	LKB and relative seriality (RS) model<br><br>LKB:<br>6m: TD50=27.8Gy, m=0.49, n=1;<br>12m: TD50=41.6Gy, m=0.45, n=1<br><br>RS:<br>6m: TD50=26.3Gy<br>12m: TD50=40.0Gy	Studied: Dmean both PGs<br><br>Included: Dmean both PGs	Fair<br>- Endpoint >=12m from RT start: yes<br>- Advanced RT techniques only, no reirradiation: yes<br>- N pts. / N events: 59/(3m: 39, 6m: 25, 12m: 10)<br>- Validation: no<br>- Prospective toxicity collection: yes<br>- Prediction probability: AUC at 12m: 0.71 (LKB) and 0.70 (S)<br>- Others: No differentiation ipsi / contra PG
Lee, 2015 [20]	67	NP	Tomotherapy	EORTC QLQ HN35 at 6m post-RT	LASSO logistic regression<br><br>NTCP = 1/(1 + exp(-s)), where<br>s= -42.149 + (age x 0.548) + (Dmean oral cavity x 0.500) + (education x coefficient) + (T stage x coefficient)	Studied: 11 clinical and 5 dosimetric parameters<br><br>Included: age, T stage, education, Dmean oral cavity	Poor<br>- Endpoint >=12m from RT start: no<br>- Advanced RT techniques only, no reirradiation: yes<br>- N pts. / N events: 54/15<br>- Validation: no<br>- Prospective toxicity collection: yes<br>- Prediction probability: AUC 0.95 (overfitting?)
Braam, 2005 [21]	52	HNC (L, FOM/OC, OP, NC, HP, NP)	3DCRT	Parotid salivary flow rate <=25% preRT flow at 6m, 12m and at least 3.5y post-RT	Lyman<br><br>6m: TD50=40Gy, m=0.33, n=1;<br>12m: TD50=42Gy, m=0.37, n=1;<br>5y: TD50=46Gy, m=0.53, n=1	Studied: sex, age, tumor site, T stage, N stage, surgery, bilateral parotid glands, stimulated parotid flow rate <25% of the preRT flow rate<br><br>Included: Dmean both	Good<br>- Endpoint >=12m from RT start: yes<br>- Advanced RT techniques only, no reirradiation: no<br>- N pts. / N events: 52/NA<br>- Validation: no<br>- Prospective toxicity collection: yes<br>- Prediction probability: NA
Miah, 2013 [22]	35	HNC (OP, HP/L, NP, Others)	IMRT	G2+ xerostomia (LENT SOMA, RTOG and XQ) at 1y post-RT	LKB<br><br>Flow: TD50=26.3Gy m=0.25, n=1;<br>LENT SOMA: TD50=28.7Gy, m=0.20, n=1	Studied & included: Dmean PG (unclear if contra or both)	Poor<br>- Endpoint >=12m from RT start: yes<br>- Advanced RT techniques only, no reirradiation: yes<br>- N pts. / N events: 35/NA<br>- Validation: no<br>- Prospective toxicity collection: yes<br>- Prediction probability: NA
Chen, 2013 [23]	31	HNC (OP,	IMRT	G3+ salivary flow at 1y and 2y	LKB	Studied: age, tumor site, chemo (yes vs. no), surgery, Dmean both PGs	Poor<br>- Endpoint >=12m from RT start: yes

Author, year Group	Number of patients	Tumor entity	RT tech-nique	Endpoint	Model	Variables studied/ included in final model	Overall quality
(same patients as [14])		HP/L, NP, Others)		post-RT	1y: TD50=43.6 Gy, m=0.18, n=1 ;<br>2y: TD50=44.5 Gy, m=0.30, n=1	Included: Dmean both PGs	- Advanced RT techniques only, no reirradiation: yes<br>- N pts. / N events: 31/NA<br>- Validation: no<br>- Prospective toxicity collection: yes<br>- Prediction probability: R^2 = 0.651 at 1y & 0.310 at 2y
Scrimger, 2004 [24]	23	HNC (OC, OP, NP, L/HP)	IMRT	Stimulated and unstimulated saliva collections every 3m for 2 years post-RT Grade2 xerostomia at 1y post-RT	Lyman<br><br>Early: TD50=12.4Gy<br>Late: TD50=43.9Gy	Studied: sex, tumor site, overall stage, def. vs. postop. RT, bi- vs. unilateral RT, Dmean both PGs, Dmean both SMGs, threshold D50<br><br>Included: Dmean both parotid	Fair<br>- Endpoint >=12m from RT start: yes<br>- Advanced RT techniques only, no reirradiation: yes<br>- N pts. / N events: 23/NA<br>- Validation: no<br>- Prospective toxicity collection: yes<br>- Prediction probability: NA
Tenhunen, 2008 [25]	20	HNC (OP, HP, L, Others)	IMRT	Unstimulated and stimulated salivary flow at 6m and 12m post-RT	Lyman<br>Scintigraphically determined dose-response models<br><br>TD50=39Gy for PG and SMG at 6 and 12 months	Studied: sex, tumor site, tumor stage, tumor size, regional LN, histology, Dmean individual glands (unclear if ipsi or contra)<br><br>Included: Dmean individual glands (unclear if ipsi or contra)	Fair<br>- Endpoint >=12m from RT start: yes<br>- Advanced RT techniques only, no reirradiation: yes<br>- N pts. / N events: 20/NA<br>- Validation: no<br>- Prospective toxicity collection: yes<br>- Prediction probability: NA
Moiseenko, 2012 [26]	66	HNC (OC, OP, NP, L/HP)	3DCRT (67%), IMRT (33%)	Parotid salivary flow rate <=25% preRT flow at 3m and 12m post-RT	LKB model<br>3m: TD50=22.2Gy, y=0.83;<br>12m: TD50=32.4Gy, y=0.97	Dmean contra parotid	Fair<br>- Endpoint >=12m from RT start: yes<br>- Advanced RT techniques only: no<br>- N pts. / N events: 66/21 at 3m and 11 at 12m<br>- Validation: no<br>- Prospective toxicity collection: yes<br>- Prediction probability: NA
Houweling, 2010 [3]	347	HNC (OP, L, OC, NP, Others, NC, HP, CUP)	3DCRT (45%), IMRT (55%)	Parotid salivary flow rate <=25% preRT flow at 12m post-RT	LKB model:<br>TD50=39.4 Gy, m=0.42, n=1.13;<br><br>Mean dose model<br>TD50=39.9 Gy, m=0.4, n=1	Dmean contra parotid	Good<br>- Endpoint >=12m from RT start: yes<br>- Advanced RT techniques only: no<br>- N pts. / N events: 347/NA at 12m<br>- Validation: no<br>- Prospective toxicity collection: yes<br>- Prediction probability: AUC 0.86 for all models
Mavroidis, 2017 [27]	32	OPC	IMRT	PRO-CTCAE at 6m & 12m	LKB at 12m (n=1):<br><br>Contra PG+SMG: TD50=26.9 Gy, m=0.65<br>Contra PG: TD50=21.9 Gy, m=0.78<br>Contra SMG: TD50=47.7 Gy, m=0.70	Dmean PG, SMG or both	Fair<br>- Endpoint >=12m from RT start: yes<br>- Advanced RT techniques only: yes<br>- N pts. / N events: 32/15 at 12m<br>- Validation: no<br>- Prospective toxicity collection: yes<br>- Prediction probability: AUC 0.67-0.73
**Dysphagia**							
Langendijk, 2009	529	HNC (L, OP, OC,	3DCRT (83%),	G2+ dysphagia (RTOG/EORTC)	Logistic regression	Studied: sex, age, tumor site, T stage, N stage, postop RT, conv. fx, acc. fx,	Fair<br>- Endpoint >=12m from RT start:

Author, year Group	Number of patients	Tumor entity	RT tech-nique	Endpoint	Model	Variables studied/ included in final model	Overall quality
[28]		HP, CUP, NP)	IMRT (10%), "Bellinzona technique (6%)	at 6m post-RT	Model parameters: unclear	chemo, RT technique, neck RT, BL dysphagia, BL weight loss<br><br>Included: tumor site, T stage, conv. fx, acc. fx, neck RT, chemo, BL weight loss,	- Advanced RT techniques only, no reirradiation: no<br>- N pts. / N events: 529 / 122<br>- Validation: yes (internal)<br>- Prospective toxicity collection: yes<br>- Prediction probability: NA<br>- Others: no dosimetric parameters included as variable; validation on diff. TPs
Wopken, 2014 [29]	427 (+183 validation)	HNC (L, OP, HP, OC, NP)	3DCRT (89%), IMRT (11%)	Feeding tube at 6m post-RT	Logistic regression<br><br>NTCP = 1/(1 + exp(-s)),<br>where s= -3.69+(T stage x 1.01) + (N stage x 0.87) + (mod. Weight loss x 0.82) + (sev. weight loss x 1.51) + (bilateral neck RT x 0.35) + (acc. RT x 0.25) + (chemo x 0.41)	Studied: Sex, age, tumor site, T stage, N stage, conv. vs. acc. fx., chemo, RT technique, BL dysphagia, neck irradiation, BL weight loss<br><br>Included: T stage, N stage, weight loss, neck irradiation, chemo	Good<br>- Endpoint >=12m from RT start: no (but feeding tube 6 months = clinically relevant)<br>- Advanced RT techniques only, no reirradiation: no<br>- N pts. / N events: 427 / 55 (training), 183 / 27 (test)<br>- Validation: no<br>- Prospective toxicity collection: yes<br>- Prediction probability: AUC 0.86 (training), 0.82 (test)
Wopken, 2014 [30]	355	HNC (OC, OP, HP/L, NP, Others)	IMRT	Feeding tube at 6m post-RT	Logistic regression<br><br>NTCP = 1/(1 + exp(-s)),<br>where s = -11.70 + (advanced T stage x 0.43) + (mod. weight loss x 0.95) + (sev. weight loss x 1.63) + (acc. RT x 1.20) + (RCT x 1.91) + (RT plus cetuximab x 0.56) + (Dmean SPC x 0.071) + (Dmean IPC x 0.034) + (Dmean PG contra x 0.006) + (Dmean cricopharyngeal muscle x 0.023)	Studied: many clinical (n=X) and dosimetric variables (n=Y), see article, table 2<br><br>Included: T stage, weight loss, acc. RT, chemo, Cetuximab, Dmean IPC, Dmean SPC, Dmean PG contra, Dmean cricopharyngeal muscle	Good<br>- Endpoint >=12m from RT start: no (but feeding tube 6 months = clinically relevant)<br>- Advanced RT techniques only, no reirradiation: yes<br>- N pts. / N events: 355/38<br>- Validation: yes (internal cross validation and external, Blanchard [6], Kanayama [31])<br>- Prospective toxicity collection: yes<br>- Prediction probability: AUC 0,88 (cross validation)
Christianen, 2012 [32]	354	HNC (L, OP, OC, HP, NP, CUP, Others)	3DCRT (62%), IMRT (38%)	G2-4 dysphagia (RTOG/EORTC) at 6m post-RT	Logistic regression<br><br>NTCP = (1 + e^-s)^-1,<br>where s= -6.09 + (Dmean SPC x 0.057) + (Dmean supraglottic larynx)<br><br>Other models for secondary endpoints: Problems swallowing liquids / soft foods / solid foods, and choking when swallowing	Studied: Sex, age, tumor site, T stage, N stage, conc. Chemo, RT technique, acc. RT, bilateral neck RT, BL dysphagia, Dmean SPC, MPC, IPC, cricopharyngeus, cerv. Esophagus, BOT, supraglottic and glottis larynx, PG both, SMG both, Deman, V50, V60 esophageal inlet muscle<br><br>Included: Dmean SPC, Dmean supraglottic larynx	Good<br>- Endpoint >=12m from RT start: no<br>- Advanced RT techniques only, no reirradiation: no<br>- N pts. / N events: 354/ NA<br>- Validation: yes (external, Blanchard [6], Hansen [33], Christianen [34])<br>- Prospective toxicity collection: yes<br>- Prediction probability: AUC 0,80
Tsai, 2017 [35]	424	HNC (OPC, NPC, L, HP, Others)	IMRT	G2+/G3+ dysphagia (CTCAE) 6 – 69m (median 33.2m) post-RT	Logistic regression<br><br>Model parameters: unclear	Studied: sex, age, tumor site, T stage, N stage, KPS, BL dysphagia, low ant. Neck fields, gEUD(a) values for SPC, MPC, IPC, total PC volume, larynx	Fair<br>- Endpoint >=12m from RT start: no<br>- Advanced RT techniques only, no reirradiation: yes<br>- N pts. / N events: 424/41 (G2+)/22(G3+)<br>- Validation: no<br>- Prospective toxicity collection: no<br>- Prediction probability: NA<br>- Others: only pts. without prior surgery and without local

Author, year Group	Number of patients	Tumor entity	RT tech-nique	Endpoint	Model	Variables studied/ included in final model	Overall quality
							failures included
Mavroidis, 2017 [27]	32	OPC	IMRT	PRO-CTCAE at 6m	LKB<br>CPC: TD50=67.2 Gy, m=0.10, n=0.01<br>SPC: TD50=62.0 Gy, m=0.10, n=0.49	Studied: Dmean CPC or SPC<br><br>Included: Dmean CPC or SPC	Fair<br>- Endpoint >=12m from RT start: no<br>- Advanced RT techniques only: yes<br>- N pts. / N events: 32/6<br>- Validation: no<br>- Prospective toxicity collection: yes<br>- Prediction probability: AUC 0.68-0.74
**Esophageal stricture**							
Mavroidis, 2003 [36]	26 + 56 controls	L, OC, OP, NP, HP, Others	3DCRT	Clinical and any grade of endoscopic esophageal stricture 1 – 40m (median 7m) post-RT, Q for controls	Relative seriality model<br><br>TD50= 68.4Gy, y=6.55, s=0.22	Studied: Dmean, Dmax. Esophagus prox. 5cm<br><br>Included: unlcear	Low<br>- Endpoint >=12m from RT start: no<br>- Advanced RT techniques only, no reirradiation: no<br>- N pts. / N events: 82 / 26<br>- Validation: no<br>- Prospective toxicity collection: no<br>- Prediction probability: ROC = 0.84, X^2 test =0.95<br>- Others: FU of control group unclear
**Dysgeusia**							
Sapir, 2016 [37]	73	OP III-IV (90% HPV+)	IMRT	Severe dysgeusia (UWQOL and HNQOL questionnaire, score >50/100) at 3m post-RT	LKB<br><br>HNQOL: D50 = 53 Gy, m=0.4, n=unclear;<br>UWQOL: D50 = 57 Gy, m=0.4, n=unclear (m value extracted from Fig 1 in manuscript)	Studied: Dmean oral cavity, anterior tongue, Dmean BOT, pretreatment taste function, time after treatment, age, sex<br><br>Included: Dmean oral cavity	Fair<br>- Endpoint >=12m from RT start: no<br>- Advanced RT techniques only, no reirradiation: yes<br>- N pts. / N events: 73/26<br>- Validation: no<br>- Prospective toxicity collection: yes<br>- Prediction probability: NA<br>- Others: Oral cavity as OAR
**Brain necrosis / Nerve palsy**							
Wang, 2019 [38]	749	NP I-IV	IMRT	G1+ temporal lobe necrosis on MRI 3.5– 75m* (median 49m) post-RT	Lasso binary regression<br><br>NTCP = 1/(1 + exp(7.36 – 0.00036 $D_{0.5cc}$ – 0.00054 D_{10}))<br>($D_{0.5cc}$/D_{10} in cGy)	Studied: sex, age, T stage, N stage, overall stage, diabetes, hypertension, Dmax, Dmin, Dmean, further DVH variables<br><br>Included: D0.5cc and D10 selected for final model	Good<br>- Endpoint >=12m from RT start: no<br>- Advanced RT techniques only, no reirradiation: yes<br>- N pts. / N events: 749/38<br>- Validation: yes (internal cross validation)<br>- Prospective toxicity collection: no<br>- Prediction probability: AUC 0.68 (testing set)
Zeng, 2015 [39]	351	NP I-IV	IMRT	G1+ temporal lobe necrosis on MRI 6 – 100m* (median 76m)	Logistic regression<br><br>P(X) = (e^((b0 + b1) x x)/(1 + e^((b0 + b1)x x),<br>“where X is the independent dose or volume variable”, and “the mode parameters b0 and b1 were determined by max. likelihood-fitting procedure”	Studied: sex, age, T stage, conc. chemo, DVH variables (n=16)<br><br>Included: D1cc	Fair<br>- Endpoint >=12m from RT start: no<br>- Advanced RT techniques only, no reirradiation: yes<br>- N pts. / N events: 351/29<br>- Validation: no<br>- Prospective toxicity collection: no<br>- Prediction probability: NA

Author, year Group	Number of patients	Tumor entity	RT tech-nique	Endpoint	Model	Variables studied/ included in final model	Overall quality
					5y: TD50=77.6 Gy		
Niyazi, 2020 [40]	179	HNC, skull base, intracranial tumors with min. dose brain/dura of 59.4Gy RBE	PSPT	G2+ brain necrosis ("symptomatic new enhancement with classicl imaging features") after min. 0.5-13.0y (median 4.4y) post-RT	Logistic regression and Mixture cure model<br><br>a=9, y50=2.5, EUD50=57.7 Gy for intracranial tumors, EUD50 = 39.5 Gy for extracranial tumors, scale λ=0.38, shape y=1.37	Studied: sex, age, conc. chemo, surgery, set of EUD indices, CTV volume, brain-GTV volume, Dmean and Dmax to brain-GTV volume<br><br>Included: tumor origin, EUD indices	Fair<br>- Endpoint >=12m from RT start: no<br>- Advanced RT techniques only, no reirradiation: yes<br>- N pts. / N events: 179/37<br>- Validation: no<br>- Prospective toxicity collection: no<br>- Prediction probability: NA
Chow, 2019 [41]	165	NPC T1-4 N0-3	IMRT	Hypoglossal nerve palsy (clinical) after min. FU 6m (median 8.1y) post-RT	Logistic regression<br><br>Model parameters: unclear	Studied: sex, age, T stage, N stage, hypoglossal canal involvement, chemo, smoking, hypertension, DM, hyperlipidaemia, connective tissue disease, T dose >70Gy, Dmax, D0.5cc, D1cc, D2cc, Dmean<br><br>Included: D1cc	Fair<br>- Endpoint >=12m from RT start: no<br>- Advanced RT techniques only, no reirradiation: yes<br>- N nerves / N events: 330/46<br>- Validation: no<br>- Prospective toxicity collection: no<br>- Prediction probability: AUC 0.83<br>- Others: all pts. min. FU 6m; healthy control min. 8y (false high rate of palsies)
Engeseth, 2020 [42]	127	HNC+Others T1-4 (NP, sinonasal, orbital, PG, skin, Others)	PT (IMPT, PSPT)	Radiation-associated MRI changes (RAIC) (T1 CE +/- T2 hyperintensity) after 6 – 97m (median 29) post-RT	Recursive partitioning analysis	Studied: Age, tumor site, T3-4, PT technique, prescribed dose, ind. chemo, conc. chemo, surgery, Dmax, D0.5cc, D1cc - D5cc in 1cc steps, V40- 70Gy(RBE) in 1Gy(RBE) steps<br><br>Included: V67Gy(RBE)	Fair<br>- Endpoint >=12m from RT start: no<br>- Advanced RT techniques only, no reirradiation: yes<br>- N pts. / N events: 127/22<br>- Validation: no<br>- Prospective toxicity collection: no<br>- Prediction probability: NA
McDonald, 2015 [43]	66	Skull base C/CS, ACC, sinonasal	PT	Temporal lobe necrosis at 6 – 96m (median 31m)	EC50 model<br><br>Y= TOP / (1 + 10^((logEC50 – X) x Hillslope)), where "TOP is the maximal risk, Hillslope the steepness of the curve, and logEC50 the irradiated temporal lobe volume in which 50% of the maximal risk is observed"	Studied: sex, age, tumor location, chemo, DM, hypertension, smoking, treatment related data, dose volume data<br><br>Included: V40 / V50 / V60 / V70	Fair<br>- Endpoint >=12m from RT start: no<br>- Advanced RT techniques only, no reirradiation: yes<br>- N temporal lobes / N events: 131 temporal lobes/12<br>- Validation: no<br>- Prospective toxicity collection: no<br>- Prediction probability: no
**Trismus**							
Morimoto, 2019 [44]	132	HNC I-IV (L, OP, HP, OC, NP)	3DCRT, IMRT (percentage unclear)	Mouth-opening <=35mm post RT or <=80% of BL at 6m post-RT	Logistic regression<br><br>NTCP = (1 + s^-s)^-1, where s= 0.494 + Dmean TMJ contra x 0.061 + (BL mouth opening x (-0.006))	Studied (univariate): sex, age, tumor site, T stage, N stage, overall stage, histology, RT technique (conv. vs. acc.), uni/bilateral neck RT, chemo, max. inter-cicsial opening at BL, Dmean medial pterygoid muscle ipsi,	Good<br>- Endpoint >=12m from RT start: no<br>- Advanced RT techniques only, no reirradiation: no<br>- N pts. / N events: 132/30<br>- Validation: no<br>- Prospective toxicity collection: yes

Author, year Group	Number of patients	Tumor entity	RT tech-nique	Endpoint	Model	Variables studied/ included in final model	Overall quality
						Dmean MM contra, Dmean TMJ contra<br><br>Included: Dmean TMJ contra, max. intercisial opening at BL	- Prediction probability: p=0.182 (Hosmer and Lemeshow test)<br>- Others: collinearity check; acc. fx in 95 pts.
Lindblom, 2014 [45]	121	HNC I-IV (OP, OC, L, HP)	3DCRT (95%), IMRT (5%)	Maximal interincisal distance (MID) <35mm after 21-127m (median 66 m) post RT-start and problems mouth-opening (EORTC H&N35 QoL scores of 3–4) at several TPs until 5y post RT-start	Logistic regression<br><br>NTCP = 1 / (1 + e^4y ^(1 – D/D50))<br><br>Model parameters for masseter:<br>MID: TD50=72.3 Gy, y50=1.04<br>QoL scores: TD50=57.2 Gy, y50=0.78	Studied: Dmean masseter, medial and lateral pterygoid muscles, TMJ (ipsi, contra and both) above mentioned mastication muscles as single structure (ipsi and contra)<br><br>Included: Models for all variables studies; best fit for ipsi masseter for both endpoints	Good<br>- Endpoint >=12m from RT start: yes for MID, unclear for QoL scores<br>- Advanced RT techniques only, no reirradiation: no<br>- N pts. / N events: 121/50<br>- Validation: yes (internal cross validation)<br>- Prospective toxicity collection: no for interincisial distance, yes for QoL<br>- Prediction probability: 0.77 and 0.73 for model with endpoint MID and QoL score 3+, respectively and ipsi masseter included;<br>- Others: 70 pts. with acc. fx
**Hypothyroidism**							
Ronjom, 2013 [46]	203	HNC (OC, OP, HP/L, NP, Others)	IMRT	TSH >4 mIU/mL at 1y and 2y post-RT	Logistic regression<br><br>NTCP = (1 + e^-s)^-1,<br>where s= -2.019 + 0.0821 x Dmean TG - 0.189 x V TG	Studied: age, sex, tumor site, stage, conc. chemo, boost technique, neck dissection after RT, BL TSH, Dmean TD, V TG<br><br>Included: Dmean TG , V TG	Good<br>- Endpoint >=12m from RT start: yes<br>- Advanced RT techniques only, no reirradiation: yes<br>- N pts. / N events: 203/35<br>- Validation: yes (external, Nowicka [47], Ronjom [48])<br>- Prospective toxicity collection: no (but objective criterion)<br>- Prediction probability: NA
Boomsma, 2012 [49]	105	HNC + skin + sarcoma (L, OP, OC, HP, SG, skin, CUP, PNS, sarcoma)	3DCRT (67%), IMRT (33%)	TSH >4mE/l and/or T4 <11pmol/l in first 2 years; median FU 2.5y (range 0.33-3.30y) post-RT	Logistic regression<br><br>NTCP = (1 + e^-s)^-1,<br>where s= 0.011 + (0.062 x Dmean TG) + (-0.19 x V TG)	Studied: sex, age, DM, conv. vs. acc. RT, RT technique, systemic therapy (chemo, Cetuximab), neck surgery, Dmean TG, Dmin. TG, Dmax TG, V20 – V60 TG, V TG<br><br>Included: Dmean TG, V TG	Good<br>- Endpoint >=12m from RT start: no<br>- Advanced RT techniques only, no reirradiation: no<br>- N pts. / N events: 105/35<br>- Validation: yes (internal bootstrapping, external, Blanchard [6], Nowicka [47], Kamal [50])<br>- Prospective toxicity collection: yes<br>- Prediction probability: AUC 0.85
Bakhshandeh, 2012 [51]	65	HNC (NP, OC, L, Ohers) with baseline normal thyroid blood tests	3DCRT	G1 hypothyroidism at 1y post-RT	Lyman, Logit, and Mean dose<br><br>Lyman:<br>TD50=44.3 Gy, m=0 .25, n=0.92<br><br>Logit EUD:<br>TD50=43.9 Gy, K-6.16, n=0.85	Studied: sex, age, tumor site, chemo, surgery, Dmean TG, Dmean pituitary, V TG<br><br>Included: Dmean pituitary	Good<br>- Endpoint >=12m from RT start: yes<br>- Advanced RT techniques only, no reirradiation: no<br>- N pts. / N events: 65/29<br>- Validation: yes (external, Nowicka [47])<br>- Prospective toxicity collection: yes<br>- Prediction probability: yes<br>- Others: no satisfactory external validation

Author, year Group	Number of patients	Tumor entity	RT tech-nique	Endpoint	Model	Variables studied/ included in final model	Overall quality
**Hearing loss**							
Cheraghi, 2017 [52]	35	HNC (PG, Sinus, NC, SG, Mandible, NP, others)	3DCRT	CTCAE v4.03 G1+ sensorial hearing loss relative to BL (pure tone audiometry) (TP unclear)	LEUD, Logit, Relative seriality (RS) model, critical volume model, and mean dose model<br><br>LEUD :<br>TD50 52.8, m=0.11, n=0.99<br><br>Logit :<br>TD50=50.4, k=10, n=0.09<br><br>Relative Seriality (RS) model:<br>TD50=52.7, y=0.68, s=0.85<br><br>Population critical volume model:<br>α=0.02, λ=0.02, N_0=11, N_{FSU}=320, D_S=108, M=0.009<br><br>Individual critical volume model:<br>γ50=10, $μ_{er}$=0.004, D50=60, δ=1.10<br><br>Mean dose model:<br>TD50=51.7Gy, m=0.14	Studied: sex, age, Dmean cochlea, V cochlea (unclear if ipsi/contra/both)<br><br>Included: unclear	Poor<br>- Endpoint >=12m from RT start: unclear<br>- Advanced RT techniques only, no reirradiation: no<br>- N ears / N events: 70/9<br>- Validation: no<br>- Prospective study: no<br>- Prediction probability: AIC a) 44.56, b) 45.87, c) 43.09, d) 50.31, e) 48.54, f) 44.64
De Marzi, 2015 [53]	140	Skull base CS/C	PT	Hearing loss >15 dB at two contiguous test frequencies >6mo post-RT	LKB (gEUD)<br>Inner ear<br><br>TD50=53.7 Gy, y=2.8, a=0.1	Studied: Dmean inner ear<br><br>Included: Dmean inner ear	Fair<br>- Endpoint >=12m from RT start: yes (median FU 26 months)<br>- Advanced RT techniques only: yes<br>- N ears/ N events: 280/73<br>- Validation: no<br>- Prospective toxicity collection: yes<br>- Prediction probability: AUC 0.86
**Tinnitus**							
Lee, 2015 [54]	211	HNC (NP, OC, HP, OP, L)	IMRT	G2+ tinnitus (LENT-SOMA) between 36 – 77m post-RT	LKB and Logistic regression<br><br>NTCP = exp(4y_{50} x (MD/TD50 – 1))/1+ exp (4y_{50} x (MD / TD50 – 1)),<br>where “MD is the mean dose to the cochlea” and “y_{50} is the normalized slope of the dose-response curve”<br><br>LKB:<br>TD50=46.52Gy, m=0.35<br><br>Logistic:	Studied: Dmean cochlea ipsi<br><br>Included: Dmean cochlea ipsi	Fair<br>- Endpoint >=12m from RT start: yes<br>- Advanced RT techniques only, no reirradiation: yes<br>- N inner ears / N events: 422/49<br>- Validation: no<br>- Prospective toxicity collection: no<br>- Prediction probability: LKB: 0.76, Logistic: 0.76<br>- Others: influence of chemotherapy not accounted for

Author, year Group	Number of patients	Tumor entity	RT tech-nique	Endpoint	Model	Variables studied/ included in final model	Overall quality
					TD50=46.31Gy, y_{50}=1.27		
**Dry eye**							
Bhandare, 2012 [55]	78	HNC + skin (orbita, sinonasal, NC, skin)	EBRT 1996 - 2000	Severe dry eye syndrome (RTOG G3/4 and/or G2/3 CTCAE) after mean of 0.9y post-RT	Logistic regression<br><br>NTCP = 1 / (1 + exp[-4y_{50}(D/D_{50} – 1)])<br>y_{50}: normalized slope at D50<br><br>QD: TD50=45 Gy<br>BID: TD50=50 Gy	Studied: sex, age, Dmax lacrimal gland, normal vs. hyperfractionation, dose per fx, chemo, dosimetric factors<br><br>Included: Dmax lacrimal gland	Fair<br>- Endpoint >=12m from RT start: no<br>- Advanced RT techniques only, no reirradiation: no<br>- N pts. / N events: 78/40<br>- Validation: yes (internal bootstrapping)<br>- Prospective toxicity collection: no<br>- Prediction probability: NA
**Laryngeal edema**							
Rancati, 2009 [56]	48	HNC (OP, CUP, NP)	IMRT	G2+ laryngeal edema (fiberoptic examination) at 15m post-RT	Lyman and Logit<br><br>Lyman:<br>TD50=47.3 Gy, m=0.23, n=1.17<br><br>Logit:<br>TD50=46.7Gy, k=7.2 Gy, n=1.41	Studied: dose fractionation, cumulative DVH without edema, cumulative DVH with edema<br><br>Included: Dmean larynx	Fair<br>- Endpoint >=12m from RT start: yes<br>- Advanced RT techniques only, no reirradiation: yes<br>- N pts. / N events: 48/25<br>- Validation: no<br>- Prospective toxicity collection: no (but objective criterion)<br>- Prediction probability: NA
**Hypopituitarism**							
De Marzi, 2015 [53]	103	Skull base CS/C	PT	Endocrine test outside normal value (dosage every 6m)	LKB (gEUD)<br><br>TD50=60.6, y=4.9, a=6.4	Studied: Dmean pituitary gland<br><br>Included: Dmean pituitary gland	Fair<br>- Endpoint >=12m from RT start: yes (median FU 26m)<br>- Advanced RT techniques only: yes<br>- N inner ears/ N events: 103/45<br>- Validation: no<br>- Prospective toxicity collection: yes<br>- Prediction probability: AUC 0.86

Abbreviations (in alphabetical order): 3DCRT: 3d conventional radiotherapy, acc.: accelerated, ACC: adenoid cystic carcinoma, AUC: area under the curve, BID: twice-a-day, BL: baseline, C: chondroma, contra: contralateral, CK: cyberknife, conv.: conventional, CPC: combined pharyngeal muscle (i.e. IPC+MPC+SPC), CS: chondrosarcoma, CUP: cancer of unknown primacy, DM: diabetes mellitus, Dmean: mean dose, DVH: dose volume histogram, EBRT: external beam radiotherapy, fx: fraction, G: grade, HNC: head neck cancer, HP: hypopharynx, IMPT: intensity modulated proton therapy, IMRT: intensity modulated radiotherapy, IPC: inferior pharyngeal constrictor, ipsi: ipsilateral, L: larynx, LKB: Lyman Kutcher Burman, m: month(s), MID: maximal intercisial distance, mod.: moderate, N: number, NA: not assessed, NC: nasal cavity, NP: nasopharynx, N stage: nodal stage, OC: oral cavity, OP: oropharynx, pts.: patients, PET IBM: positron emission tomography imaging biomarker, PG: parotid gland, prev.: previous, PSPT: pencil beam scanned proton therapy, PT: proton therapy, QD: once-a-day, QoL: quality of life questionnaire, RCT: radiochemotherapy, RT: radiotherapy, SBRT: stereotactic radiotherapy, sev.: severe, SMG: submandibular gland, SPC: superior pharyngeal muscle, T: tumor, TD50: dose predicting 50% risk of complication, TG: thyroid gland, TMJ: temporomandibular joint, TP: time point, TSH: thyroid stimulating hormone, V: volume, VMAT: volumetric modulated arc therapy, vs.: versus, w: week(s), y: year(s)
