# Supplementary material for: NTCP Modeling of Late Effects for Head and Neck Cancer: A Systematic Review
Source: Int J Part Ther. 2021 Jun 25;8(1):95–107. doi: 10.14338/20-00092 (PMC8270107; doi:10.14338/20-00092)
Supplement: Supplementary file 2 [file ijpt-08-01-26_s02.pdf]

Supplementary Material

References of Supplementary Table I

1. van Dijk LV, Brouwer CL, van der Schaaf A, Burgerhof JGM, Beukinga RJ, Langendijk JA, Sijtsema NM, Steenbakkers R. CT image biomarkers to improve patient-specific prediction of radiation-induced xerostomia and sticky saliva. *Radiother Oncol.* 2017;122:185-91.
2. Dijkema T, Raaijmakers CP, Ten Haken RK, Roesink JM, Braam PM, Houweling AC, Moerland MA, Eisbruch A, Terhaard CH. Parotid gland function after radiotherapy: the combined michigan and utrecht experience. *Int J Radiat Oncol Biol Phys.* 2010;78:449-53.
3. Houweling AC, Philippens ME, Dijkema T, Roesink JM, Terhaard CH, Schilstra C, Ten Haken RK, Eisbruch A, Raaijmakers CP. A comparison of dose-response models for the parotid gland in a large group of head-and-neck cancer patients. *Int J Radiat Oncol Biol Phys.* 2010;76:1259-65.
4. Dijkema T, Terhaard CH, Roesink JM, Braam PM, van Gils CH, Moerland MA, Raaijmakers CP. Large cohort dose-volume response analysis of parotid gland function after radiotherapy: intensity-modulated versus conventional radiotherapy. *Int J Radiat Oncol Biol Phys.* 2008;72:1101-9.
5. Beetz I, Schilstra C, van der Schaaf A, van den Heuvel ER, Doornaert P, van Luijk P, Vissink A, van der Laan BF, Leemans CR, Bijl HP, Christianen ME, Steenbakkers RJ, Langendijk JA. NTCP models for patient-rated xerostomia and sticky saliva after treatment with intensity modulated radiotherapy for head and neck cancer: the role of dosimetric and clinical factors. *Radiother Oncol.* 2012;105:101-6.
6. Blanchard P, Wong AJ, Gunn GB, Garden AS, Mohamed ASR, Rosenthal DI, Crutison J, Wu R, Zhang X, Zhu XR, Mohan R, Amin MV, Fuller CD, Frank SJ. Toward a model-based patient selection strategy for proton therapy: External validation of photon-derived normal tissue complication probability models in a head and neck proton therapy cohort. *Radiother Oncol.* 2016;121:381-6.
7. Beetz I, Schilstra C, Burlage FR, Koken PW, Doornaert P, Bijl HP, Chouvalova O, Leemans CR, de Bock GH, Christianen ME, van der Laan BF, Vissink A, Steenbakkers RJ, Langendijk JA. Development of NTCP models for head and neck cancer patients treated with three-dimensional conformal radiotherapy for xerostomia and sticky saliva: the role of dosimetric and clinical factors. *Radiother Oncol.* 2012;105:86-93.
8. Beetz I, Schilstra C, van Luijk P, Christianen ME, Doornaert P, Bijl HP, Chouvalova O, van den Heuvel ER, Steenbakkers RJ, Langendijk JA. External validation of three dimensional conformal radiotherapy based NTCP models for patient-rated xerostomia and sticky saliva among patients treated with intensity modulated radiotherapy. *Radiother Oncol.* 2012;105:94-100.
9. van Dijk LV, Noordzij W, Brouwer CL, Boellaard R, Burgerhof JGM, Langendijk JA, Sijtsema NM, Steenbakkers R. (18)F-FDG PET image biomarkers improve prediction of late radiation-induced xerostomia. *Radiother Oncol.* 2018;126:89-95.
10. Gabrys HS, Buettner F, Sterzing F, Hauswald H, Bangert M. Parotid gland mean dose as a xerostomia predictor in low-dose domains. *Acta Oncol.* 2017;56:1197-203.
11. Roesink JM, Moerland MA, Hoekstra A, Van Rijk PP, Terhaard CH. Scintigraphic assessment of early and late parotid gland function after radiotherapy for head-and-neck cancer: a prospective study of dose-volume response relationships. *Int J Radiat Oncol Biol Phys.* 2004;58:1451-60.
12. Lee TF, Liou MH, Huang YJ, Chao PJ, Ting HM, Lee HY, Fang FM. LASSO NTCP predictors for the incidence of xerostomia in patients with head and neck squamous cell carcinoma and nasopharyngeal carcinoma. *Sci Rep.* 2014;4:6217.
13. Lee TF, Chao PJ, Ting HM, Chang L, Huang YJ, Wu JM, Wang HY, Horng MF, Chang CM, Lan JH, Huang YY, Fang FM, Leung SW. Using multivariate regression model with least absolute shrinkage and selection operator (LASSO) to predict the

incidence of Xerostomia after intensity-modulated radiotherapy for head and neck cancer. *PLoS One.* 2014;9:e89700.
14. Lee TF, Chao PJ, Wang HY, Hsu HC, Chang P, Chen WC. Normal tissue complication probability model parameter estimation for xerostomia in head and neck cancer patients based on scintigraphy and quality of life assessments. *BMC Cancer.* 2012;12:567.
15. Roesink JM, Moerland MA, Battermann JJ, Hordijk GJ, Terhaard CH. Quantitative dose-volume response analysis of changes in parotid gland function after radiotherapy in the head-and-neck region. *Int J Radiat Oncol Biol Phys.* 2001;51:938-46.
16. Hey J, Setz J, Gerlach R, Vordermark D, Gernhardt CR, Kuhnt T. Effect of Cisplatin on parotid gland function in concomitant radiochemotherapy. *Int J Radiat Oncol Biol Phys.* 2009;75:1475-80.
17. Eisbruch A, Ten Haken RK, Kim HM, Marsh LH, Ship JA. Dose, volume, and function relationships in parotid salivary glands following conformal and intensity-modulated irradiation of head and neck cancer. *Int J Radiat Oncol Biol Phys.* 1999;45:577-87.
18. van Dijk LV, Thor M, Steenbakkers R, Apte A, Zhai TT, Borra R, Noordzij W, Estilo C, Lee N, Langendijk JA, Deasy JO, Sijtsema NM. Parotid gland fat related Magnetic Resonance image biomarkers improve prediction of late radiation-induced xerostomia. *Radiother Oncol.* 2018;128:459-66.
19. Marzi S, Iaccarino G, Pasciuti K, Soriani A, Benassi M, Arcangeli G, Giovinazzo G, Benassi M, Marucci L. Analysis of salivary flow and dose-volume modeling of complication incidence in patients with head-and-neck cancer receiving intensity-modulated radiotherapy. *Int J Radiat Oncol Biol Phys.* 2009;73:1252-9.
20. Lee TF, Liou MH, Ting HM, Chang L, Lee HY, Wan Leung S, Huang CJ, Chao PJ. Patient- and therapy-related factors associated with the incidence of xerostomia in nasopharyngeal carcinoma patients receiving parotid-sparing helical tomotherapy. *Sci Rep.* 2015;5:13165.
21. Braam PM, Roesink JM, Moerland MA, Raaijmakers CP, Schipper M, Terhaard CH. Long-term parotid gland function after radiotherapy. *Int J Radiat Oncol Biol Phys.* 2005;62:659-64.
22. Miah AB, Gulliford SL, Clark CH, Bhide SA, Zaidi SH, Newbold KL, Harrington KJ, Nutting CM. Dose-response analysis of parotid gland function: what is the best measure of xerostomia? *Radiother Oncol.* 2013;106:341-5.
23. Chen WC, Lai CH, Lee TF, Hung CH, Liu KC, Tsai MF, Wang WH, Chen H, Fang FM, Chen MF. Scintigraphic assessment of salivary function after intensity-modulated radiotherapy for head and neck cancer: correlations with parotid dose and quality of life. *Oral Oncol.* 2013;49:42-8.
24. Scrimger RA, Stavrev P, Parliament MB, Field C, Thompson H, Stavreva N, Fallone BG. Phenomenologic model describing flow reduction for parotid gland irradiation with intensity-modulated radiotherapy: evidence of significant recovery effect. *Int J Radiat Oncol Biol Phys.* 2004;60:178-85.
25. Tenhunen M, Collan J, Kouri M, Kangasmaki A, Heikkonen J, Kairemo K, Makitie A, Joensuu H, Saarilahti K. Scintigraphy in prediction of the salivary gland function after gland-sparing intensity modulated radiation therapy for head and neck cancer. *Radiother Oncol.* 2008;87:260-7.
26. Moiseenko V, Wu J, Hovan A, Saleh Z, Apte A, Deasy JO, Harrow S, Rabuka C, Muggli A, Thompson A. Treatment planning constraints to avoid xerostomia in head-and-neck radiotherapy: an independent test of QUANTEC criteria using a prospectively collected dataset. *Int J Radiat Oncol Biol Phys.* 2012;82:1108-14.
27. Mavroidis P, Price A, Fried D, Kostich M, Amdur R, Mendenhall W, Liu C, Das S, Marks LB, Chera B. Dose-volume toxicity modeling for de-intensified chemo-radiation therapy for HPV-positive oropharynx cancer. *Radiother Oncol.* 2017;124:240-7.
28. Langendijk JA, Doornaert P, Rietveld DH, Verdonck-de Leeuw IM, Leemans CR, Slotman BJ. A predictive model for swallowing dysfunction after curative radiotherapy in head and neck cancer. *Radiother Oncol.* 2009;90:189-95.

29. Wopken K, Bijl HP, van der Schaaf A, Christianen ME, Chouvalova O, Oosting SF, van der Laan BF, Roodenburg JL, Leemans CR, Slotman BJ, Doornaert P, Steenbakkers RJ, Verdonck-de Leeuw IM, Langendijk JA. Development and validation of a prediction model for tube feeding dependence after curative (chemo-) radiation in head and neck cancer. *PLoS One.* 2014;9:e94879.
30. Wopken K, Bijl HP, van der Schaaf A, van der Laan HP, Chouvalova O, Steenbakkers RJ, Doornaert P, Slotman BJ, Oosting SF, Christianen ME, van der Laan BF, Roodenburg JL, Leemans CR, Verdonck-de Leeuw IM, Langendijk JA. Development of a multivariable normal tissue complication probability (NTCP) model for tube feeding dependence after curative radiotherapy/chemo-radiotherapy in head and neck cancer. *Radiother Oncol.* 2014;113:95-101.
31. Kanayama N, Kierkels RGJ, van der Schaaf A, Steenbakkers R, Yoshioka Y, Nishiyama K, Fujii T, Ogawa K, Langendijk JA, Teshima T. External validation of a multifactorial normal tissue complication probability model for tube feeding dependence at 6months after definitive radiotherapy for head and neck cancer. *Radiother Oncol.* 2018;129:403-8.
32. Christianen ME, Schilstra C, Beetz I, Muijs CT, Chouvalova O, Burlage FR, Doornaert P, Koken PW, Leemans CR, Rinkel RN, de Bruijn MJ, de Bock GH, Roodenburg JL, van der Laan BF, Slotman BJ, Verdonck-de Leeuw IM, Bijl HP, Langendijk JA. Predictive modelling for swallowing dysfunction after primary (chemo)radiation: results of a prospective observational study. *Radiother Oncol.* 2012;105:107-14.
33. Hansen CR, Friborg J, Jensen K, Samsoe E, Johnsen L, Zukauskaite R, Grau C, Maare C, Johansen J, Primdahl H, Bratland A, Kristensen CA, Andersen M, Eriksen JG, Overgaard J. NTCP model validation method for DAHANCA patient selection of protons versus photons in head and neck cancer radiotherapy. *Acta Oncol.* 2019;58:1410-5.
34. Christianen ME, van der Schaaf A, van der Laan HP, Verdonck-de Leeuw IM, Doornaert P, Chouvalova O, Steenbakkers RJ, Leemans CR, Oosting SF, van der Laan BF, Roodenburg JL, Slotman BJ, Bijl HP, Langendijk JA. Swallowing sparing intensity modulated radiotherapy (SW-IMRT) in head and neck cancer: Clinical validation according to the model-based approach. *Radiother Oncol.* 2016;118:298-303.
35. Tsai CJ, Jackson A, Setton J, Riaz N, McBride S, Leeman J, Kowalski A, Happersett L, Lee NY. Modeling Dose Response for Late Dysphagia in Patients With Head and Neck Cancer in the Modern Era of Definitive Chemoradiation. *JCO Clin Cancer Inform.* 2017;1:1-7.
36. Mavroidis P, Laurell G, Kraepelien T, Fernberg JO, Lind BK, Brahme A. Determination and clinical verification of dose-response parameters for esophageal stricture from head and neck radiotherapy. *Acta Oncol.* 2003;42:865-81.
37. Sapir E, Tao Y, Feng F, Samuels S, El Naqa I, Murdoch-Kinch CA, Feng M, Schipper M, Eisbruch A. Predictors of Dysgeusia in Patients With Oropharyngeal Cancer Treated With Chemotherapy and Intensity Modulated Radiation Therapy. *Int J Radiat Oncol Biol Phys.* 2016;96:354-61.
38. Wang J, Miao Y, Ou X, Wang X, He X, Shen C, Ying H, Hu W, Hu C. Development and validation of a model for temporal lobe necrosis for nasopharyngeal carcinoma patients with intensity modulated radiation therapy. *Radiat Oncol.* 2019;14:42.
39. Zeng L, Huang SM, Tian YM, Sun XM, Han F, Lu TX, Deng XW. Normal Tissue Complication Probability Model for Radiation-induced Temporal Lobe Injury after Intensity-modulated Radiation Therapy for Nasopharyngeal Carcinoma. *Radiology.* 2015;276:243-9.
40. Niyazi M, Niemierko A, Paganetti H, Sohn M, Schapira E, Goldberg S, Adams J, Kim V, Oh KS, Hwang WL, Lu HM, Belka C, Busse PM, Loeffler JS, Shih HA. Volumetric and actuarial analysis of brain necrosis in proton therapy using a novel mixture cure model. *Radiother Oncol.* 2020;142:154-61.
41. Chow JCH, Cheung KM, Au KH, Zee BCY, Lee J, Ngan RKC, Lee AWM, Yiu HHY, Li KWS, Leung AKC, Chan JCH, Lee FKH, Wong KH. Radiation-induced hypoglossal

nerve palsy after definitive radiotherapy for nasopharyngeal carcinoma: Clinical predictors and dose-toxicity relationship. *Radiother Oncol.* 2019;138:93-8.

42. Engeseth GM, Stieb S, Mohamed ASR, He R, Stokkevag CH, Brydoy M, Fuller CD, Garden AS, Rosenthal DI, Phan J, Morrison WH, Reddy JP, Wu R, Zhang X, Frank SJ, Brandon Gunn G. Outcomes and patterns of radiation associated brain image changes after proton therapy for head and neck skull base cancers. *Radiother Oncol.* 2020;151:119-25.
43. McDonald MW, Linton OR, Calley CS. Dose-volume relationships associated with temporal lobe radiation necrosis after skull base proton beam therapy. *Int J Radiat Oncol Biol Phys.* 2015;91:261-7.
44. Morimoto M, Bijl HP, A VDS, Xu CJ, Steenbakkers R, Chouvalova O, Yoshioka Y, Teshima T, Langendijk JA. Development of Normal Tissue Complication Probability Model for Trismus in Head and Neck Cancer Patients Treated With Radiotherapy: The Role of Dosimetric and Clinical Factors. *Anticancer Res.* 2019;39:6787-98.
45. Lindblom U, Garskog O, Kjellen E, Laurell G, Levring Jaghagen E, Wahlberg P, Zackrisson B, Nilsson P. Radiation-induced trismus in the ARTSCAN head and neck trial. *Acta Oncol.* 2014;53:620-7.
46. Ronjom MF, Brink C, Bentzen SM, Hegedus L, Overgaard J, Johansen J. Hypothyroidism after primary radiotherapy for head and neck squamous cell carcinoma: normal tissue complication probability modeling with latent time correction. *Radiother Oncol.* 2013;109:317-22.
47. Nowicka Z, Tomasik B, Papis-Ubych A, Bibik R, Graczyk L, Latusek T, Rutkowski T, Wyka K, Fijuth J, Schoenfeld JD, Chalubinska-Fendler J, Fendler W. Radiation-Induced Hypothyroidism in Patients with Oropharyngeal Cancer Treated with IMRT: Independent and External Validation of Five Normal Tissue Complication Probability Models. *Cancers (Basel).* 2020;12.
48. Ronjom MF, Brink C, Bentzen SM, Hegedus L, Overgaard J, Petersen JB, Primdahl H, Johansen J. External validation of a normal tissue complication probability model for radiation-induced hypothyroidism in an independent cohort. *Acta Oncol.* 2015;54:1301-9.
49. Boomsma MJ, Bijl HP, Christianen ME, Beetz I, Chouvalova O, Steenbakkers RJ, van der Laan BF, Wolffenbuttel BH, Oosting SF, Schilstra C, Langendijk JA. A prospective cohort study on radiation-induced hypothyroidism: development of an NTCP model. *Int J Radiat Oncol Biol Phys.* 2012;84:e351-6.
50. Kamal M, Peeler CR, Yepes P, Mohamed ASR, Blanchard P, Frank S, Chen L, Jethanandani A, Kuruvilla R, Greiner B, Harp J, Granberry R, Mehta V, Rock C, Hutcheson K, Cardenas C, Gunn GB, Fuller C, Mirkovic D. Radiation-Induced Hypothyroidism After Radical Intensity Modulated Radiation Therapy for Oropharyngeal Carcinoma. *Adv Radiat Oncol.* 2020;5:111-9.
51. Bakhshandeh M, Hashemi B, Mahdavi SR, Nikoofar A, Vasheghani M, Kazemnejad A. Normal tissue complication probability modeling of radiation-induced hypothyroidism after head-and-neck radiation therapy. *Int J Radiat Oncol Biol Phys.* 2013;85:514-21.
52. Cheraghi S, Nikoofar A, Bakhshandeh M, Khoei S, Farahani S, Abdollahi H, Mahdavi SR. Normal tissue complication probability modeling of radiation-induced sensorineural hearing loss after head-and-neck radiation therapy. *Int J Radiat Biol.* 2017;93:1327-33.
53. De Marzi L, Feuvret L, Boule T, Habrand JL, Martin F, Calugaru V, Fournier-Bidoz N, Ferrand R, Mazal A. Use of gEUD for predicting ear and pituitary gland damage following proton and photon radiation therapy. *Br J Radiol.* 2015;88:20140413.
54. Lee TF, Yeh SA, Chao PJ, Chang L, Chiu CL, Ting HM, Wang HY, Huang YJ. Normal tissue complication probability modeling for cochlea constraints to avoid causing tinnitus after head-and-neck intensity-modulated radiation therapy. *Radiat Oncol.* 2015;10:194.

55. Bhandare N, Moiseenko V, Song WY, Morris CG, Bhatti MT, Mendenhall WM. Severe dry eye syndrome after radiotherapy for head-and-neck tumors. *Int J Radiat Oncol Biol Phys.* 2012;82:1501-8.
56. Rancati T, Fiorino C, Sanguineti G. NTCP modeling of subacute/late laryngeal edema scored by fiberoptic examination. *Int J Radiat Oncol Biol Phys.* 2009;75:915-23.
